# Lignin concentrations in phloem and outer bark are not associated with resistance to mountain pine beetle among high elevation pines

**DOI:** 10.1371/journal.pone.0250395

**Published:** 2021-09-23

**Authors:** David N. Soderberg, Bethany Kyre, Pierluigi Bonello, Barbara J. Bentz

**Affiliations:** 1 Wildland Resources Department, Utah State University, Logan, Utah, United States of America; 2 Ecology Center, Utah State University, Logan, Utah, United States of America; 3 Department of Entomology, University of Kentucky, Lexington, KY, United States of America; 4 Department of Plant Pathology, The Ohio State University, Columbus, OH, United States of America; 5 US Forest Service, Rocky Mountain Research Station, Logan, Utah, United States of America; University of Catania, ITALY

## Abstract

A key component in understanding plant-insect interactions is the nature of host defenses. Research on defense traits among *Pinus* species has focused on specialized metabolites and axial resin ducts, but the role of lignin in defense within diverse systems is unclear. We investigated lignin levels in the outer bark and phloem of *P*. *longaeva*, *P*. *balfouriana*, and *P*. *flexilis*; tree species growing at high elevations in the western United States known to differ in susceptibility to mountain pine beetle (*Dendroctonus ponderosae*; MPB). *Pinus longaeva* and *P*. *balfouriana* are attacked by MPB less frequently than *P*. *flexilis*, and MPB brood production in *P*. *longaeva* is limited. Because greater lignification of feeding tissues has been shown to provide defense against bark beetles in related genera, such as *Picea*, we hypothesized that *P*. *longaeva* and *P*. *balfouriana* would have greater lignin concentrations than *P*. *flexilis*. Contrary to expectations, we found that the more MPB-susceptible *P*. *flexilis* had greater phloem lignin levels than the less susceptible *P*. *longaeva* and *P*. *balfouriana*. No differences in outer bark lignin levels among the species were found. We conclude that lignification in *Pinus* phloem and outer bark is likely not adaptive as a physical defense against MPB.

## Introduction

Bark beetles (Coleoptera: Curculionidae, Scolytinae) are forest disturbance agents globally and include many tree-killing species [1]. Overcoming tree defenses is a central challenge for bark beetles which feed on living phloem and requires the destruction of tree vascular tissue for offspring survival. Tree defenses provide protection against insect attack, thereby maintaining the functional integrity of two subcortical high-fitness-value tissue types: phloem, which is responsible for transport and distribution of photosynthate produced in leaves and needles; and xylem, which provides structural support and functions in translocation of water and dissolved minerals from roots to the rest of the tree. Both tissue types are involved in the production and/or storage of defensive structures and compounds, and thus play a crucial role in defense against bark beetles [2,3] and their fungal mutualists [4–6].

The mountain pine beetle (MPB) (*Dendroctonus ponderosae* Hopkins, Coleoptera: Curculionidae, Scolytinae) is an ecologically and economically significant bark beetle with an extensive distribution across western North America [7,8]. While the majority of *Pinus* species are considered MPB hosts [9], successful MPB attacks on *P*. *longaeva* (Great Basin bristlecone pine) and *P*. *balfouriana* (foxtail pine) are rare [10], relative to the commonly attacked *P*. *flexilis* (limber pine) [11–13]. In addition, MPB displays aversion to *P*. *longaeva* in both field [14] and laboratory settings [15], and extremely few MPB offspring emerge from manually infested *P*. *longaeva* relative to *P*. *flexilis* [16]. *P*. *longaeva* and *P*. *balfouriana* also have dense sapwood and heartwood and possess high concentrations of constitutive specialized metabolite defense compounds relative to co-occurring *P*. *flexilis* [10].

Specialized metabolites as well as anatomical structures are fundamental in conifer defense. They can be expressed constitutively or upregulated upon attack as needed to maximize the economy of available resources [17–20]. Variation among and within conifer species in chemical [10,21,22] and anatomical defenses [23,24] is well known and hypothesized to reflect resistance to multiple bark beetle species [25,26]. Specialized metabolites include low molecular weight (LMW) compounds (e.g., terpenes and their derivatives, phenolics) that can be toxic to attacking bark beetle adults [27–29] and their eggs and larvae [30], and inhibit the propagation of fungal symbionts [31]. Anatomical defenses are structural elements (e.g., resin ducts, lignified stone cells) that can deter invading insects by providing physical and chemical barriers to nutrient-rich tissues [20,32,33].

Lignin, a fundamental plant structural element, is the second most naturally abundant biopolymer in plant cell walls, after cellulose [34,35]. Lignin is deposited in the secondary cell wall of all vascular plants [36,37] where it provides rigidity for structural stability and impermeability for more efficient water transport [38], as well as structural resilience against abiotic stressors [39–42]. Lignin also plays a role in tree defense, where it can increase resistance to degradation by microorganisms [43–45], and provide protection against pathogenic fungi [46,47] and bacteria [48]. Cell wall lignification also confers tree resistance against herbivory in the form of sublethal chemical defenses (i.e., antifeedant or antinutritional) [49,50] and direct physical defenses [51].

In the family *Pinaceae*, sclerenchyma cells of the phloem occur as large stone cells that are primarily comprised of lignin [32,52,53]. Increased stone cell frequencies within the phloem of Sitka spruce (*Picea sitchensis* Bongard) were associated with decreased spruce weevil (*Pissodes strobi* Peck) growth rate, survival, and fecundity, and disruption of larval establishment [53–56]. Decreased growth rate and survival of great spruce bark beetle (*Dendroctonus micans* Kugelann) larvae were also associated with increased lignin concentrations [32,57] and naturally occurring compounds originating from lignin were found to have antifeedant effects on the large pine weevil, *Hylobius abietis* (L.) [58]. Moreover, lignin synthase genes were found to be more prevalent in spruce that were -resistant to *P*. *strobi* [53]. Because lignified tissue is difficult to chew and digest [32,53,59] it can reduce nutritional quality and nutrient bioavailability [51,60,61] by preventing adequate feeding and increasing mandibular wear [32].

The genus *Pinus*, specifically, is known to have evolved various defensive strategies against phloem-feeding insects, such as specialized metabolites [62–64] and resin ducts [20,65,66], both of which show high variability within and among *Pinus* species [67,68]. Little is known, however, of the role of lignin as a constitutive defensive mechanism against bark beetle attacks in tree species growing at high elevations in the western United States that are at increasing risk due to climate change. We attempted to fill this gap by quantifying lignin in the outer bark (i.e., rhytidome) and phloem of co-occurring *P*. *longaeva*, *P*. *balfouriana* and *P*. *flexilis* from multiple sites and compared lignin concentrations within and among species and between the two tissue types. We hypothesized that the more MPB-resistant *P*. *longaeva* and *P*. *balfouriana* would have greater lignin concentrations than co-occurring *P*. *flexilis*.

## Methods

### Study locations and tree sampling

**Fig 1:**
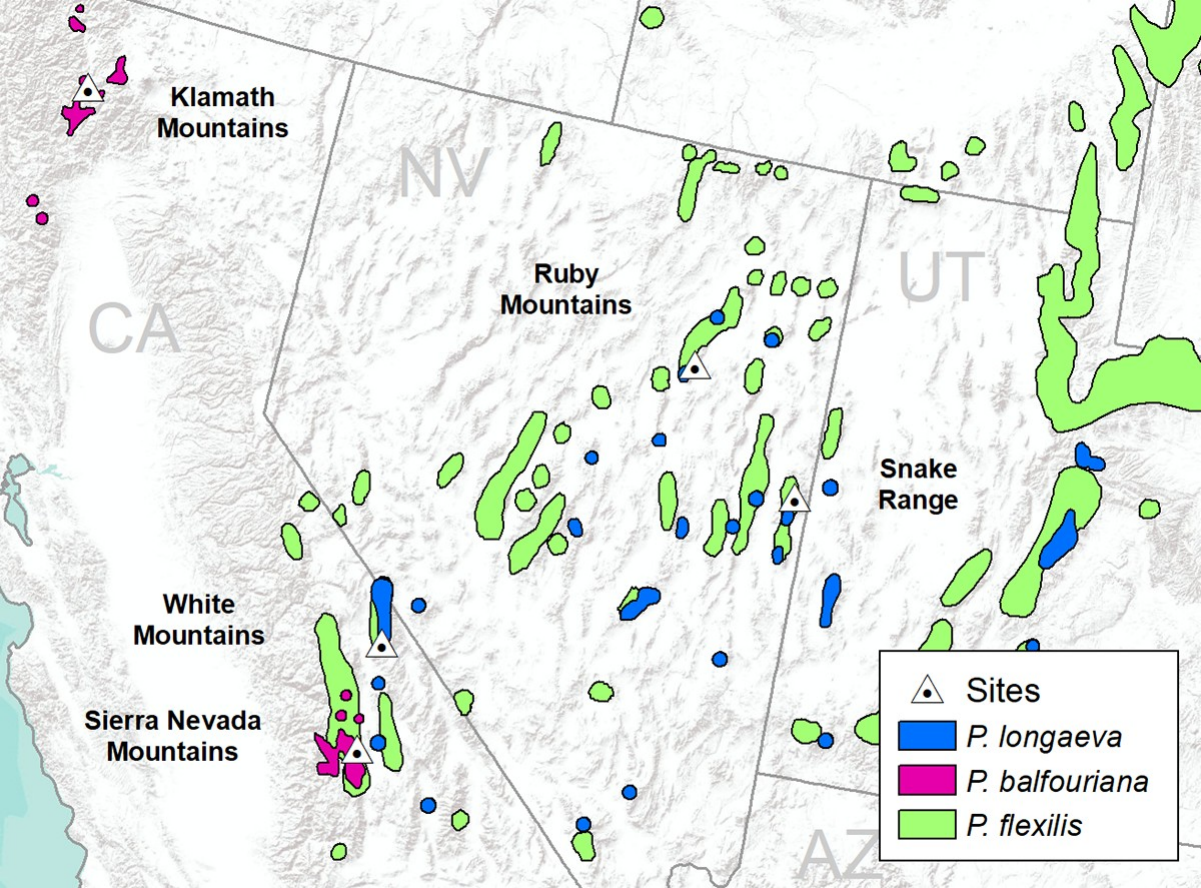
Distributions of Great Basin bristlecone pine (*Pinus longaeva*), foxtail pine (*P*. *balfouriana*), and limber pine (*P*. *flexilis*), and sample site locations (see Table 1). Pine distributions are based on Little (1971) [69].

Between June and September 2016, trees were sampled at five sites across the ranges of *P*. *longaeva* and *P*. *balfouriana*, four of them in stands with co-occurring *P*. *flexilis* (Fig 1; Table 1). Four of the five sites were also sampled by Bentz et al. (2017) [10], allowing a comparison with results from that study. Equal numbers of *P*. *longaeva* and *P*. *flexilis* trees were sampled at three geographically separated locations, and equal numbers of *P*. *balfouriana* and *P*. *flexilis* were sampled at the Sierra Nevada site. At the Klamath site *P*. *flexilis* was not present, and only *P*. *balfouriana* was sampled. At each site 15 live trees of each species were sampled, and diameter at breast height (DBH, ~ 1.5 m above ground) ranged from 30–46 cm. Study sites without signs of MPB or pathogen activity were chosen to avoid an influence of induced defenses. Permission for sampling was obtained through the Inyo, Klamath, Sierra Nevada and Humboldt-Toiyabe National Forests.

**Table 1:** Site locations (see Fig 1) and stand metrics including species sampled, number of phloem and bark samples analyzed, and mean ± standard error of DBH (diameter breast height).

Site	*Pinus* species	Latitude	Longitude	Elevation (m)	Number of samples (phloem/bark)	DBH (cm)
Klamath Mountains, CA, USA	*P*. *balfouriana----------*	41.21700	-122.79700	1965	15/14	36.9 ± 1.13
Snake Range, NV, USA	*P*. *longaeva*	39.28849	-114.20270	3048	15/13	40.7 ± 0.81
	*P*. *flexilis*				15/10	37.9 ± 1.02
Ruby Mountains, NV, USA	*P*. *longaeva*	40.19808	-115.55583	2932	15/8	40.5 ± 1.01
	*P*. *flexilis*				15/13	38.9 ± 0.82
Sierra Nevada Mountains, CA, USA	*P*. *balfouriana*	36.49560	-118.17834	3046	14/12	37.7 ± 1.24
	*P*. *flexilis*				15/15	37.8 ± 1.26
White Mountains, CA, USA	*P*. *longaeva*	37.39338	-118.19019	3127	14/13	38.5 ± 1.15
	*P*. *flexilis*				14/5	37.8 ± 0.93

To assess lignin levels (mg/g fresh weight) in outer bark and phloem, trees were sampled by boring into the tree at breast height with a 1” diameter circular hole saw (Milwaukee^TM^). Four samples were taken on the north, south, west, and east aspects of the tree trunk and pooled to account for potential within-tree variation. Upon tissue removal, phloem thickness (mm) was measured from the north and south aspect samples. Outer bark and phloem tissues were then separated and placed immediately in a sealed vial in a cooler with dry ice for transport to the Rocky Mountain Research Station (Logan, UT) for cold storage (-40°C).

### Lignin extraction

In the laboratory experiments, outer bark and phloem samples were prepared for lignin extraction using a ceramic mortar and pestle to grind tissue samples in liquid nitrogen. Tissues were ground to a fine powder and placed in vials for lignin extraction. The mortar and pestle were cleaned with 95% ethanol between each tissue sample. Lignin was extracted from the outer bark and phloem tissues using thioglycolic acid digestion in a modification of the method of Bruce and West (1989) [70], as described by Bonello et al. (1993) [71]. Spectral absorbance of phloem lignin samples (n = 135) was measured at 280 nm using a NanoDrop™ 3300 Fluorospectrometer (Thermofisher Scientific) with a 1:4 dilution in NaOH against a standard curve of pure spruce lignin (Sigma-Aldrich) at 0, 18, 45, 90, and 360 micrograms/mL. The spectral absorbance of outer bark lignin (n = 103) was measured under the same parameters using 1:64 dilution. All phloem samples were assessed as pure and free from contamination, although thirty-two outer bark samples were removed from analysis due to residual phenolic compound contamination (S1 Fig). In addition, three outliers, consisting of a single phloem sample from each species (2% of total samples), exhibited lignin concentration > 6-fold the standard deviation for each species. As the outer bark contained remarkably higher lignin concentrations than the phloem, we removed these three outliers out of caution for potential tissue contamination. Adjusted sample sizes for outer bark and phloem samples are shown in Table 1.

### Statistical analysis

Differences among tree species in phloem and outer bark lignin concentrations, phloem thickness, and DBH were assessed with a hierarchical mixed effect analysis of variance (ANOVA), that accounts for variation among sites, using the package “lme4” [72] in R version 4.0.0 [73]. Multiple comparisons among sites were assessed using the package “multcomp” [74]. Linear regression (package “lme4”) was used to assess the relationships between phloem and outer bark lignin concentrations, phloem lignin concentration and phloem thickness, DBH and phloem thickness, DBH and phloem lignin concentration, and DBH and outer bark lignin concentration.

## Results

**Fig 2:**
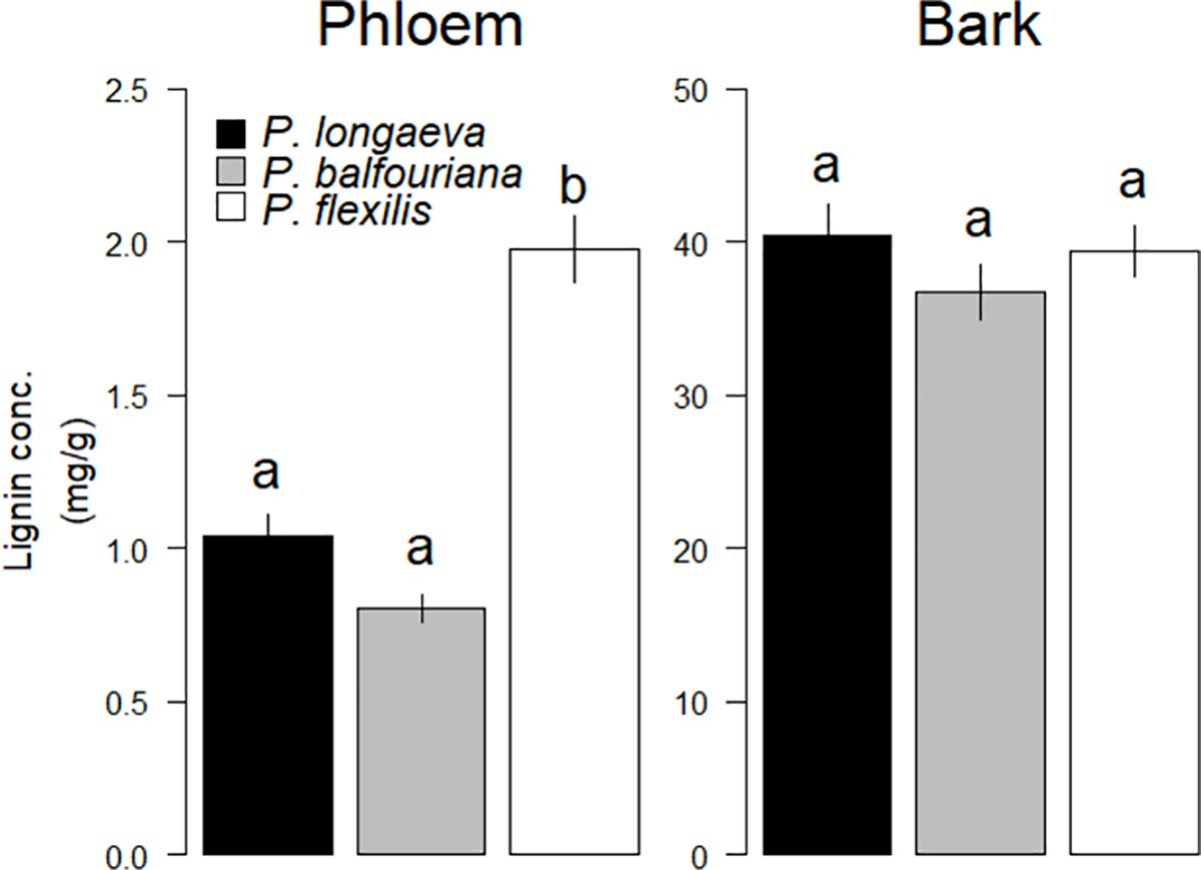
Phloem and bark lignin concentratons (± standard error) in *P*. *longaeva*, *P*. *balfouriana*, and *P*. *flexilis* averaged across all sites. Different letters (i.e., a,b) denote statistically significant differences among species means (p < 0.05). See Table 2 for statistics.

**Fig 3:**
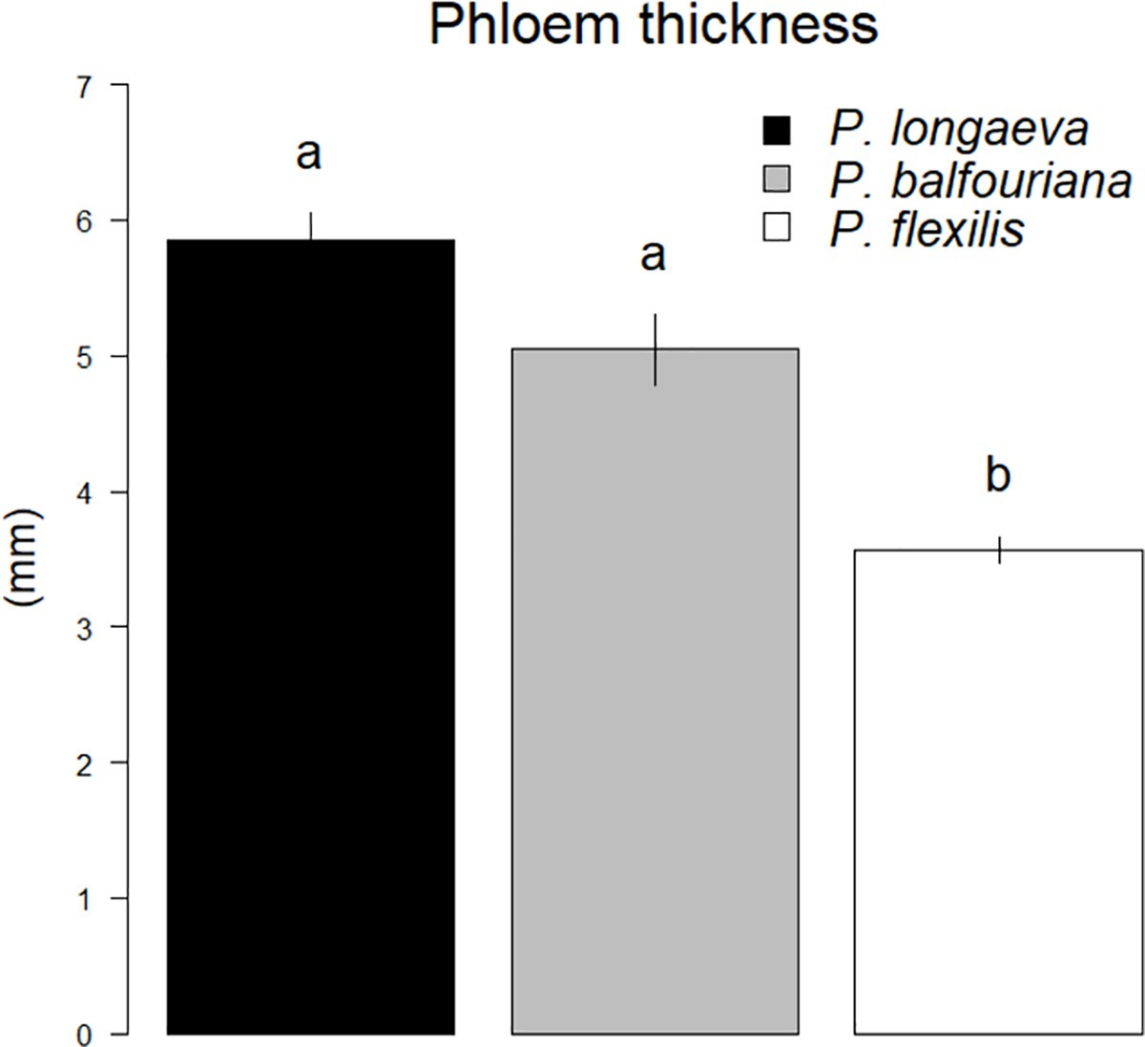
Phloem thickness (± standard error) in *P*. *longaeva*, *P*. *balfouriana*, and *P*. *flexilis*, averaged across all sites (see Fig 1; Table 1). Different letters (i.e., a,b) denote statistically significant differences among species means (p < 0.05). See Table 2 for statistics.

Phloem lignin concentrations did not differ between *P*. *longaeva* and *P*. *balfouriana*, but, contrary to our hypotheses, *P*. *flexilis* had significantly higher (~2-fold) phloem lignin concentrations than the other two species (Fig 2; Table 2). We found no differences among the species in outer bark lignin concentrations (Fig 2; Table 2). *P*. *flexilis* had thinner phloem than both *P*. *longaeva* and *P*. *balfouriana*, but there were no differences in phloem thickness between *P*. *longaeva* and *P*. *balfouriana* (Fig 3; Table 2). *P*. *flexilis* trees with thicker phloem tended to have lower phloem lignin levels, but we found no relationship between phloem thickness and phloem lignin levels in *P*. *longaeva* or *P*. *balfouriana* (Table 3). We also found no relationship between phloem thickness and outer bark lignin levels in any species (Table 3). *P*. *flexilis* and *P*. *balfouriana* were generally smaller than *P*. *longaeva* (Table 2), although DBH had no effect on phloem or lignin concentrations in any of the species (Table 3). There was also no significant relationship between phloem and outer bark lignin concentrations among trees, although *P*. *balfouriana* with more phloem lignin tended to have less outer bark lignin (Table 3). There were no significant differences among the sites in phloem lignin concentration for any species (*P*. *flexilis*: p > 0.238; *P*. *longaeva*: p > 0.095; *P*. *balfouriana*: p = 0.101), although *P*. *flexilis* outer bark lignin concentration differed at two sites (S1 Table).

**Table 2:** Model estimates testing for species differences in diameter at breast height (DBH; cm), phloem thickness (mm), and phloem and bark lignin concentrations (mg/g FW) among *P*. *flexilis*, *P*. *balfouriana*, and *P*. *longaeva*.

	DBH		Phloem thickness		Phloem lignin		Bark lignin		
	Est. (95%CI)	p	Est. (95%CI)	p	Est. (95%CI)	p		Est. (95%CI)	p	
*P*. *flexilis* vs *P*. *balfouriana*	0.71 (-1.76, 3.19)	0.780	-1.83 (-2.73, -0.03)	**<0.0001**	0.91 (0.47, 1.33)	**<0.0001**		3.04 (-4.91, 11.0)	0.641	
*P*. *flexilis* vs *P*. *longaeva*	-2.19 (-4.40, 0.01)	0.052	-2.27 (-2.89, -1.65)	**<0.0001**	1.03 (0.75, 1.32)	**<0.0001**		-1.54 (-7.58, 4.51)	0.821	
*P*. *balfouriana* vs *P*. *longaeva*	-2.91 (-5.38, -0.43)	**0.016**	-0.44 (-1.48, 0.61)	0.584	0.13 (-0.36, 0.62)	0.744		-4.57 (-13.5, 4.32)	0.448	

Effect size (Est.) and 95% confidence interval (95%CI) estimates between comparison samples are shown. P-values (p) describe the likelihood of statistical difference with values < 0.05 presented in bold.

**Table 3:** Modeled linear regression coefficients (i.e., slope) testing for the relationship between phloem thickness (mm) and DBH (diameter at breast height, cm), phloem thickness and phloem lignin concentrations (mg/g FW), phloem lignin concentrations and DBH, bark lignin concentrations and DBH, and phloem and bark thickness within *P*. *flexilis*, *P*. *balfouriana*, and *P*. *longaeva* across sites.

	Phloem thickness × DBH		Phloem thickness ×Phloem lignin conc.		Phloem lignin conc. × DBH		Bark lignin conc. × DBH		Phloem lignin conc. × Bark lignin conc.	
	Est. (95% CI)	p	Est. (95% CI)	p	Est. (95% CI)	p	Est. (95% CI)	p	Est. (95% CI)	p
*P*. *flexilis*	0.14 (-0.02, 0.31)	0.097	-0.35 (-0.65, -0.03)	**0.037**	-0.03 (-0.10, 0.03)	0.310	0.14 (-0.95, 0.74)	0.739	0.02 (-0.01, 0.04)	0.201
*P*. *balfouriana*	0.03 (-0.23, 0.31)	0.812	-0.32 (-2.38, 2.03)	0.772	-0.01 (-0.03, 0.02)	0.493	-0.24 (-1.06, 0.57)	0.565	-0.01 (-0.02, -0.00)	0.055
*P*. *longaeva*	-0.10 (-0.37, 0.15)	0.448	-0.11 (-1.18, 0.88)	0.826	0.01 (-0.02, 0.05)	0.403	0.15 (-0.84, 1.18)	0.765	-0.00 (-0.01, 0.01)	0.892

Effect size (Est.) and 95% confidence interval (95% CI) estimates between comparison samples are shown. P-values (p) presented describe the likelihood of statistical difference with values < 0.05 presented in bold.

## Discussion

Contrary to our expectations, *P*. *flexilis* exhibited the highest levels of constitutive phloem lignin relative to co-occurring *P*. *longaeva* and *P*. *balfouriana*¸ although there were no differences among the species in outer bark lignin. We also found no consistent relationship between phloem and outer bark lignin concentrations at the tree species level. Because *P*. *flexilis* is considered more susceptible to MPB and produces greater numbers of offspring than *P*. *longaeva* and *P*. *balfouriana*, our results suggest that in these species constitutive lignin may not function as a direct defense against MPB attack or brood production. Our findings are similar to previous studies that showed phloem lignification did not differ among ash species (*Fraxinus spp*.) with varying resistance to the phloephagous emerald ash borer (*Agrilus planipennis* Fair.) [75,76]. Although constitutive phloem lignin, as measured in our study, may not provide a significant defense, methyl jasmonate-induced lignification of *F*. *americana* and *F*. *pennsylvanica* phloem/outer bark was associated with resistance to the emerald ash borer [77]. The potential for induced lignification to act as an active defense in the *Pinus* species we sampled has not been investigated and should be part of future studies.

*Pinus flexilis* has consistently been found to have less constitutive and induced LMW specialized metabolites (i.e., terpenes and their derivations) than other species, including *P*. *longaeva* and *P*. *balfouriana* at the sites sampled for this study [10], *P*. *contorta* and *P*. *ponderosa* [78], and the closely related bristlecone species *P*. *aristata* (Soderberg et al. in review). Although interspecific differences in selective pressure may have led to differences in investment in phloem specialized metabolite defenses [10,79,80], our findings suggest an inverse relationship between lignification and phloem chemical defenses that are known to provide defense against bark beetles [26,31]. In our study, *P*. *flexilis* had thinner phloem, but greater lignin concentrations and absolute abundance than *P*. *longaeva* and *P*. *balfouriana*, the latter two having thicker phloem. Moreover, *P*. *flexilis* with the thickest phloem had the lowest lignin concentrations, further suggesting a negative relationship between phloem thickness and lignification. That outer bark lignin concentrations did not differ among the tree species but phloem concentrations did, suggest that lignification within the phloem may be under different selective pressures relative to outer bark. Trait associations and underlying mechanisms facilitating phloem lignification may be unique to the functions of nutrient transport or defense against invading bacteria or pathogens [81].

In summary, if defense against bark beetle attack were a strong selective driver for higher lignification in *Pinus*, higher lignin levels would be expected within both outer bark and phloem tissues of species considered less susceptible to MPB. This expectation is supported by prior research in *Picea* that was focused on species that are generally not considered primary mortality agents of mature trees, including *Pissodes* larva that feed in terminal buds [53–56,69], *H*. *abietis* that girdle seedlings [58], and the base-feeding *D*. *micans* [32,57]. MPB is a bole feeder that often kills mature trees. Our results showing that the more frequently MPB-attacked *P*. *flexilis* had greater phloem lignin concentrations relative to the less MPB-susceptible *P*. *longaeva* and *P*. *balfouriana* suggest that the defensive function of lignin may be dependent on the plant tissue consumed and aggressiveness of the insect. We also found that the species with the greatest constitutive phloem lignin concentrations, *P*. *flexilis*, was previously found to produce lower levels of constitutive LMW specialized metabolites than the other two species. While increased tissue lignification may have an additive effect with specialized metabolites on host defenses against MPB, there may be metabolic tradeoffs that are not accounted for between LMW specialized metabolites and lignin. Therefore, greater lignification within feeding tissues does not appear to be generally adaptive as a defense against MPB. Moreover, interspecific differences in phloem but not outer bark lignin concentrations highlight that the benefits and costs of lignification in *Pinus* are likely specific to phloem tissue. High elevation *Pinus* species are increasingly threatened by MPB as a result of warming temperatures. Our results enhance the important knowledge base of defense strategies employed by MPB-susceptible high elevation *Pinus*.

## Supporting information

S1 Fig Lignin extracts of phloem and outer bark samples. All phloem samples were clear and colorless and therefore assumed pure (left vial). Outer bark samples were assumed to be pure when clear and colorless to light pink (right vial), but incompletely digested and/or contaminated when dark red (middle vial).
(JPG)

S1 Table Model estimates testing for differences in phloem and bark lignin concentrations (mg/g FW) among sample sites of *P*. *flexilis*, *P*. *longaeva*, and *P*. *balfouriana* (see Table 1, Fig 1). Effect size (Est.) and 95% confidence interval (95% CI) estimates between comparison samples are shown. P-values (p) presented describe the likelihood of statistical difference with values < 0.05 presented in bold.
(DOCX)

## References

[1] Biedermann PH, Müller J, Grégoire JC, Gruppe A, Hagge J, Hammerbacher A, et al. Bark beetle population dynamics in the Anthropocene: challenges and solutions. Trends Ecol. Evol. 2019; 34: 914–924. doi: 10.1016/j.tree.2019.06.002 31262532

[2] Atkins MD. Behavioural variation among scolytids in relation to their habitat. Can. Entomol. 1966; 98: 285–288. 10.4039/Ent98285-3.

[3] Raffa KF, Phillips TW, Salom SM. Strategies and mechanisms of host colonization by bark beetles. In: Schowalter T, Filip G (eds) Beetle-pathogen interactions in conifer forests. Academic Press, New York; 1993. pp 103–128.

[4] Ballard RG, Walsh MA, Cole WE. The penetration and growth of blue-stain fungi in the sapwood of lodgepole pine attacked by mountain pine-beetle. Can. J. Bot. 1984; 62:1724–1729. 10.1139/b84-233.

[5] Långstrom B, Solheim H, Hellqvist C, Gref R. Effects of pruning young Scots pines on host vigor and susceptibility to *Leptographium Wingfieldii* and *Ophiostoma Minus*, two blue-stain fungi associated with *Tomicus Piniperda*. Eur. J. For. Pathol. 1993; 23: 400–415. 10.1111/j.1439-0329.1993.tb00820.x.

[6] Wullschleger SD, McLaughlin SB, Ayres MP. High-resolution analysis of stem increment and sap flow for loblolly pine trees attacked by southern pine beetle. Can. J. For. Res. 2004; 34: 2387–2393. 10.1139/x04-118.

[7] Cooke CJ, Carroll AL. Predicting the risk of mountain pine beetle spread to eastern pine forests: Considering uncertainty in uncertain times. For. Ecol. Manag. 2017; 396: 11–25. 10.1016/j.foreco.2017.04.008.

[8] Dowle EJ, Bracewell RR, Pfender ME, Mock KE, Bentz BJ, Ragland GJ. Reproductive isolation and environmental adaptation shape the phylogeography of mountain pine beetle (*Dendroctonus ponderosae*). Mol. Ecol. 2017; 26: 6071–6084. doi: 10.1111/mec.14342 29116665

[9] Wood SL. The bark and ambrosia beetles of North and Central America (Coleoptera: Scolytidae), a taxonomic monograph. Gt. Basin Nat. Mem. 1982; 6: 1–1359.

[10] Bentz BJ, Hood SM, Hansen EM, Vandygriff JC, Mock KE. Defense traits in the long-lived Great Basin bristlecone pine and resistance to the native herbivore mountain pine beetle. New Phytol. 2017; 213: 611–624. doi: 10.1111/nph.14191 27612209 PMC5213150

[11] Langor DW. Host effects on the phenology, development, and mortality of field populations of the mountain pine beetle, *Dendroctonus ponderosae* Hopkins (Coleoptera: Scolytidae). Can. Entomol. 1989; 121: 149–157. 10.4039/Ent121149-2.

[12] Cleaver CM, Jacobi WR, Burns KS, Means RE. Limber pine in the central and southern Rocky Mountains: stand conditions and interactions with blister rust, mistletoe, and bark beetles. For. Ecol. Manag. 2015; 358: 139–153. 10.1016/j.foreco.2015.09.010.

[13] Bentz BJ, Hansen M, Vandygriff JC, Stephens S, Soderberg DN. Rocky Mountain bristlecone pine (*P. aristata*) is a confirmed host to mountain pine beetle (*Dendroctonus ponderosae*). West. N. Am. Nat. 2021; 81: 19–26.

[14] Eidson EL, Mock KE, Bentz BJ. Mountain pine beetle host selection behavior confirms high resistance in Great Basin bristlecone pine. For. Ecol. Manag. 2017; 402: 12–20. 10.1016/j.foreco.2017.06.034.

[15] Gray C, Runyon JB, Jenkins MJ, Guinta AD. Mountain pine beetles use volatile cues to locate host limber pine and avoid non-host Great Basin bristlecone pine. PLoS ONE 2015; 10: e0135752. doi: 10.1371/journal.pone.0135752 26332317 PMC4558103

[16] Eidson EL, Mock KE, Bentz BJ. Low offspring survival in mountain pine beetle infesting the resistant Great Basin bristlecone pine supports the preference-performance hypothesis. PloS ONE. 2018; 13: e0196732. doi: 10.1371/journal.pone.0196732 29715269 PMC5929522

[17] Luchi N, Ma R, Capretti P, Bonello P. Systemic induction of traumatic resin ducts and resin flow in Austrian pine by wounding and inoculation with *Sphaeropsis sapinea* and *Diplodia scrobiculata*. Planta. 2005; 221: 75–84. doi: 10.1007/s00425-004-1414-3 15843966

[18] Steppuhn A, Baldwin IT. Induced Defenses and the Cost-Benefit Paradigm. In: Schaller A. (eds) Induced Plant Resistance to Herbivory. 2008, Springer, Dordrecht. doi: 10.1111/j.1365-294X.2008.03862.x

[19] Cipollini DF, Heil M. Costs and benefits of induced resistance to herbivores and pathogens in plants. CAB Reviews: Persp. Ag., Vet. Sci., Nutr., Nat. Res. 2010; 5: 1–25. doi: 10.1079/PAVSNNR20105005

[20] Hood S, Sala A, Heyerdahl EK, Boutin M. Low-severity fire increases tree defense against bark beetle attacks. Ecology 2015; 96: 1846–1855. doi: 10.1890/14-0487.1 26378307

[21] Zavarin E, Cool LG, Snajberk K. Geographical variability of *Pinus flexilis* xylem monoterpenes. Biochem. Syst. Ecol. 1993; 21: 381–387. 10.1016/0305-1978(93)90029-Q.

[22] Taft S, Najar A, Godbout J, Bousquet J, Erbilgin N. Variations in foliar monoterpenes across the range of jack pine reveal three widespread chemotypes: implications to host expansion of invasive mountain pine beetle. Front. Plant Sci. 2015; 6: 1–12. doi: 10.3389/fpls.2015.00001 26042134 PMC4436562

[23] Ferrenberg S, Kane J, Mitton J. Resin duct characteristics associated with tree resistance to bark beetles across lodgepole and limber pines. Oecologia. 2014; 174: 1283–1292. doi: 10.1007/s00442-013-2841-2 24305863

[24] Moreira X, Zas R, Solla A, Sampedro L. Differentiation of persistent anatomical defensive structures is costly and determined by nutrient availability and genetic growth-defence constraints. Tree Physiol. 2015; 35: 112–123. doi: 10.1093/treephys/tpu106 25595753

[25] Phillips MA, Croteau R. Resin based defenses in conifers. Trends Plant Sci. 1999; 4: 184–190. doi: 10.1016/s1360-1385(99)01401-6 10322558

[26] Keeling CI, Bohlmann J. Genes, enzymes and chemicals of terpenoid diversity in the constitutive and induced defence of conifers against insects and pathogens. New Phytol. 2006; 170: 657–675. doi: 10.1111/j.1469-8137.2006.01716.x 16684230

[27] Cook SP, Hain FP. Toxicity of host monoterpenes to *Dendroctonus frontalis* and *Ips calligraphus* (Coleoptera: Scolytidae), J. Entomol. Sci. 1988; 23: 287–292. 10.18474/0749-8004-23.3.287.

[28] Chiu CC, Keeling CI, Bohlmann J. Toxicity of pine monoterpenes to mountain pine beetle. Sci. Rep. 2017; 7: 6–13. doi: 10.1038/s41598-017-00059-1 28821756 PMC5562797

[29] Reid ML, Sekhon JK, LaFramboise LM. Toxicity of monoterpene structure, diversity and concentration to mountain pine beetles, *Dendroctonus ponderosae*: beetle traits matter more. J. Chem. Ecol. 2017; 43: 1–24. doi: 10.1007/s10886-016-0812-x 28258318

[30] Raffa KR, Berryman AA. Physiological aspects of lodgepole pine wound responses to a fungal symbiont of the mountain pine beetle *Dendroctonus ponderosae* (Coleoptera: Scolytidae). Can. Entomol. 1983; 115: 723–734. 10.4039/Ent115723-7.

[31] Franceschi VR, Krokene P, Christiansen E, Krekling T. Anatomical and chemical defenses of conifer bark against bark beetles and other pests. New Phytol. 2005; 167: 353–376. doi: 10.1111/j.1469-8137.2005.01436.x 15998390

[32] Wainhouse D, Cross DJ, Howell RS. The role of lignin as a defence against the Spruce Bark Beetle *Dendroctonus micans*: Effect on Larvae and Adults. Oecologia. 1990; 85: 257–265. doi: 10.1007/BF00319411 28312565

[33] Krokene P, Nagy NE, Krekling T. Traumatic resin ducts and polyphenolic parenchyma cells in conifers. In: Schaller A. (Ed.), Induced Plant Resistance to Herbivory. Springer, Berlin, 2008. pp. 147–169. doi: 10.1093/treephys/28.1.29

[34] Freudenberg K, Neish AC. Constitution and Biosynthesis of Lignin. New York, NY: Springer-Verlag Inc.; 1968.

[35] Lewis GN, Sarkanen S. Lignin and lignan biosynthesis. ACS Symposium Series. 1998; 697: 436.

[36] Sarkar P, Bosneaga E, Auer M. Plant cell walls throughout evolution: towards a molecular understanding of their design principles. J. Exp. Bot. 2009; 60: 3615–3635. doi: 10.1093/jxb/erp245 19687127

[37] Bonawitz ND, Chapple C. The Genetics of Lignin Biosynthesis: Connecting Genotype to Phenotype. Ann. Rev. Gen. 2010; 44: 337–363. doi: 10.1146/annurev-genet-102209-163508 20809799

[38] Voelker SL, Lachenbruch B., Meinzer FC, Kitin P, Strauss SH. Transgenic poplars with reduced lignin show impaired xylem conductivity, growth efficiency, and survival. Plant Cell Environ. 2010; 34: 655–668. 10.1111/j.1365-3040.2010.02270.x. 21309794

[39] Boerjan W, Ralph J, Baucher M. Lignin biosynthesis. Annu, Rev. Plant Biol. 2003; 54: 519–46. 10.1146/annurev.arplant.54.031902.134938. 14503002

[40] Rubin EM. Genomics of cellulosic biofuels. Nature 2008; 454: 841–845. doi: 10.1038/nature07190 18704079

[41] Moura JC, Bonine CA, Viana JD, Dornelos MC, Mazzafera P. Abiotic and biotic stresses and changes in the lignin content and composition in plants. J. Integr. Plant Biol. 2010; 52: 360–376. doi: 10.1111/j.1744-7909.2010.00892.x 20377698

[42] Sadeghifar H, Ragauskas A. Lignin as a UV light blocker–A Review. Polymers. 2020; 12: 1134. doi: 10.3390/polym12051134 32429134 PMC7284897

[43] Kirk TK, Higachi T, Chang H. Lignin biodegradation: summary and perspectives. In Lignin Biodegradation: Microbiology, Chemistry, and potential applications. II., 1979. pp. 235–45.

[44] Boudet AM, Lapierre C, Grima-Pettenati J. Tansley review no. 80: Biochemistry and molecular biology of lignification. New Phytol. 1995; 129: 203–236. doi: 10.1111/j.1469-8137.1995.tb04292.x 33874561

[45] Weng J, Chapple C. The origin and evolution of lignin biosynthesis. New Phytol. 2010; 187: 273–285. doi: 10.1111/j.1469-8137.2010.03327.x 20642725

[46] Nicholson RL, Hammerschmidt R. Phenolic compounds and their role in disease resistance. Annu. Rev. Phytopathol. 1992; 30: 369–389. 10.1146/annurev.py.30.090192.002101.

[47] Bonello P., and Blodgett J. T. 2003. *Pinus nigra-Sphaeropsis sapinea* as a model pathosystem to investigate local and systemic effects of fungal infection of pines. Physiol. Mol. Plant Pathol. 63: 249–261. 10.1016/j.pmpp.2004.02.002.

[48] Zhang SH, Yang Q, Ma RA. *Erwinia carotovora* ssp. carotovora infection induced “defense lignin” accumulation and lignin biosynthetic gene expression in Chinese cabbage (*Brassica rapa* L. *ssp. pekinensis*). J. Integr. Plant Biol. 2007; 49: 993–1002. 10.1111/j.1672-9072.2007.00478.x.

[49] Bhuiyan NH, Selvaraj G, Wei Y, King J. Role of lignification in plant defense. Plant Signal. Behav. 2009; 4: 158–59. doi: 10.4161/psb.4.2.7688 19649200 PMC2637510

[50] Sattler SE, Funnell-Harris D. Modifying lignin to improve bioenergy feedstocks: strengthening the barrier against pathogens. Front. Plant. Sci. 2013. doi: 10.3389/fpls.2013.00070 23577013 PMC3617363

[51] Johnson MT, Smith SD, Rausher MD. Plant sex and the evolution of plant defenses against herbivores. Proc. Natl. Acad. Sci. 2009; 106: 79–84. doi: 10.1073/pnas.0811468106 19617572 PMC2775293

[52] Tao S, Khanizadeh S, Zhang H, Zhang S. Anatomy, ultrastructure and lignin distribution of stone cells in two *Pyrus* species. Plant Sci. 2009; 76: 413–419. 10.1016/j.plantsci.2008.12.011.

[53] Whitehill JGA, Henderson H, Strong W, Jaquish B, Bohlmann J. Function of Sitka spruce stone cells as a physical defence against white pine weevil. Plant Cell Environ. 2016; 39: 2545–2556. doi: 10.1111/pce.12810 27478980

[54] Grau M, Alfaro RI, Brown G. Bark traits related to resistance to the white pine weevil in selected Sitka spruce families. Canadian Forest Service and B.C. Ministry of Forestry, Pacific Forestry Centre, Victoria, B.C. Unpublished file rep.; 2001.

[55] King JN, Alfaro RI, Lopez MG, Akker LV. Resistance of Sitka spruce (*Picea sitchensis* (Bong.) Carr.) to white pine weevil (*Pissodes strobi* Peck): characterizing the bark defence mechanisms of resistant populations. Forestry. 2011; 84: 83–91. 10.1093/forestry/cpq047.

[56] Whitehill JGA, Yuen MS, Henderson H, Madilao L, Kshatriya K, Bryan J, et al. Functions of stone cells and oleoresin terpenes in the conifer defense syndrome. New Phytol. 2019; 221: 1503–1517. doi: 10.1111/nph.15477 30216451

[57] Wainhouse D, Ashburner R, Ward E, Boswell, R. The Effect of Lignin and Bark Wounding on Susceptibility of Spruce Trees to *Dendroctonus micans*. J. Chem. Ecol. 1998; 24: 1551–1561. 10.1023/A:1020915901756.

[58] Borg-Karlson AK, Nordlander G, Mudalige A, Nordenhem H, Unelius CR. Antifeedants in the Feces of the Pine Weevil *Hylobius abietis*: Identification and Biological Activity. J. Chem. Ecol. 2006; 32: 943–957. doi: 10.1007/s10886-006-9050-y 16739015

[59] Wainhouse D, Ashburner R. The influence of genetic and environmental factors on a quantitative defensive trait in Spruce. Funct. Ecol. 1996; 10: 137–143. 10.2307/2390272.

[60] Swain T. Tannins and Lignin. In: Rosenthal GA, Janzen DH (eds) Herbivores: Their interaction with secondary plant metabolites. Academic Press, New York, 1979. pp 657–682.

[61] Rhoades MJC. The physiological significant of plant phenolic compounds. In C.F. Van Sumere and P.J. Lea (eds.), Annual Proceedings of the Phytochemical Society of Europe, Vol. 25: the Biochemistry of Plant Phenolics, 1985. pp. 99–117. Clarendon Press, Oxford.

[62] Villari C, Faccoli M, Battisti A, Bonello P, Marini L (2014) Testing phenotypic trade-offs in the chemical defence strategy of Scots pine under growth-limiting field conditions. Tree Physiol. 2014; 34: 919–930. doi: 10.1093/treephys/tpu063 25194142

[63] Keefover-Ring K, Trowbridge A, Mason C, Raffa KF. Rapid induction of multiple terpenoid groups by ponderosa pine in response to bark beetle-associated fungi. J. Chem. Ecol. 2016; 42: 1–12. doi: 10.1007/s10886-015-0659-6 26662358

[64] Raffa KF, Mason CJ, Bonello P, Cook S, Erbilgin N, Keefover-Ring K, et al. (2017) Defence syndromes in lodgepole–whitebark pine ecosystems relate to degree of historical exposure to mountain pine beetles. Plant Cell Environ. 2017; 40: 1791–1806. doi: 10.1111/pce.12985 28543133

[65] Kane JM, Kolb TE. Importance of resin ducts in reducing ponderosa pine mortality from bark beetle attack. Oecologia. 2010; 164: 601–609. doi: 10.1007/s00442-010-1683-4 20556621

[66] Gaylord ML, Kolb TE, Pockman WT, Plaut JA, Yepez EA, Macalady AK, et al. Drought predisposes piñon–juniper woodlands to insect attacks and mortality. New Phytol. 2013; 198: 567–578. doi: 10.1111/nph.12174 23421561

[67] López-Goldar X., Villari C, Bonello P, Borg-Karlson AK, Grivet D, Zas R, et al. Inducibility of Plant Secondary Metabolites in the Stem Predicts Genetic Variation in Resistance Against a Key Insect Herbivore in Maritime Pine. Front. Plant Sci. 2018; 9: 1651. doi: 10.3389/fpls.2018.01651 30519249 PMC6258960

[68] López-Goldar X., Villari C, Bonello P, Borg-Karlson AK, Grivet D, Sampedro L, et al. Genetic variation in the constitutive defensive metabolome and its inducibility are geographically structured and largely determined by demographic processes in maritime pine. J. Ecol. 2019; 107: 2464–2477. 10.1111/1365-2745.13159.

[69] Little E. Atlas of United States tree. Vol. 1 Conifers and important hardwoods. Misc. pub. 1146. Washington, DC, USA: US Department of Agriculture, Forest Service, 1971.

[70] Bruce R, West C. Elicitation of lignin biosynthesis and isoperoxidase activity by pectic fragments in suspension cultures of castor bean. Plant Physiol. 1989; 91: 889–897. doi: 10.1104/pp.91.3.889 16667153 PMC1062092

[71] Bonello P, Heller W, Sandermann H. Ozone effects on root-disease susceptibility and defence responses in mycorrhizal and non-mycorrhizal seedlings of Scots pine (*Pinus sylvestris* L.). New Phytol. 1993; 124: 653–663. doi: 10.1111/j.1469-8137.1993.tb03855.x 33874431

[72] Bates D, Mächler M, Bolker B, Walker S. Fitting Linear Mixed-Effects Models Using lme4. Journal of Statistical Software. 2015; 67: 1–48. doi: 10.18637/jss.v067.i01

[73] R Core Team R. A Language and Environment for Statistical Computing (R Foundation for Statistical Computing, Vienna, 2020); http://www.R-project.org/).

[74] Hothorn T, Bretz F, Westfall P. Simultaneous Inference in General Parametric Models. Biom. J. 2008; 50: 346–363. doi: 10.1002/bimj.200810425 18481363

[75] Cipollini D, Wang Q, Whitehill J, Powell J, Bonello P, Herms D. Distinguishing defensive characteristics in the phloem of ash species resistant and susceptible to emerald ash borer. J. Chem. Ecol. 2011; 37: 450–459. doi: 10.1007/s10886-011-9954-z 21537902

[76] Whitehill JGA, Opiyo S, Koch J, Herms D, Cipollini D, Bonello P. Interspecific comparison of constitutive ash phloem phenolic chemistry reveals compounds unique to Manchurian ash, a species resistant to emerald ash borer. J. Chem. Ecol. 2012; 38: 499–511. doi: 10.1007/s10886-012-0125-7 22588569

[77] Whitehill JGA, Rigsby C, Cipollini D, Herms DA, Bonello P. Decreased emergence of emerald ash borer from ash treated with methyl jasmonate is associated with induction of general defense traits and the toxic phenolic compound verbascoside. Oecologia. 2014; 176: 1047–1059. doi: 10.1007/s00442-014-3082-8 25231373

[78] Ferrenberg S, Langenhan JM, Loskot SA, Rozal LM and Mitton JB. Resin monoterpene defenses decline within three widespread species of pine (Pinus) along a 1530-m elevational gradient. Ecosphere. 2017; 8: 1–18. doi: 10.1002/ecs2.2052 29552374 PMC5854398

[79] Raffa KF, Powell EN, Townsend PA. Temperature-driven range expansion of an irruptive insect heightened by weakly coevolved plant defenses. Proc. Nat. Acad. Sci. 2013; 110: 2193–2198. doi: 10.1073/pnas.1216666110 23277541 PMC3568305

[80] Erbilgin N, Ma C, Whitehouse C, Shan B, Najar A, Evenden M. Chemical similarity between historical and novel host plants promotes range and host expansion of the mountain pine beetle in a naïve host ecosystem. New Phytol. 2014; 201: 940–950. doi: 10.1111/nph.12573 24400902

[81] Vance CP, Kirk TK, Sherwood RT. Lignification as a mechanism of disease resistance. Ann. Rev. Phytopath. 1980; 18:259–288. 10.1146/annurev.py.18.090180.001355.

