# Supplementary figures and images for: Lignin concentrations in phloem and outer bark are not associated with resistance to mountain pine beetle among high elevation pines
Source: PLoS One. 2021 Sep 23;16(9):e0250395. doi: 10.1371/journal.pone.0250395 (PMC8460017; doi:10.1371/journal.pone.0250395)

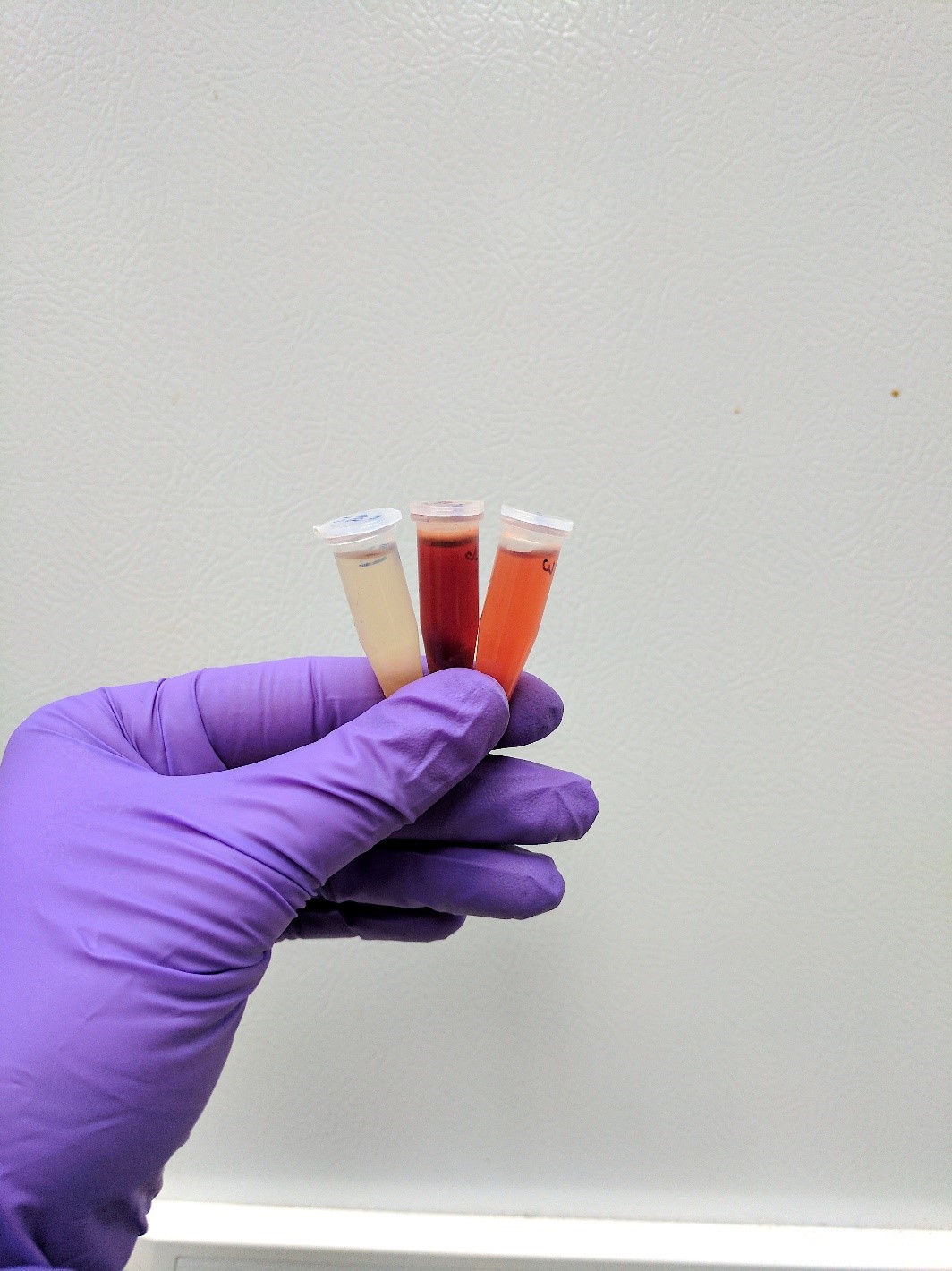

Supplement: S1 Fig — All phloem samples were clear and colorless and therefore assumed pure (left vial). Outer bark samples were assumed to be pure when clear and colorless to light pink (right vial), but incompletely digested and/or contaminated when dark red (middle vial). (JPG) [file pone.0250395.s001.jpg]
