# Supplementary material for: Lignin concentrations in phloem and outer bark are not associated with resistance to mountain pine beetle among high elevation pines
Source: PLoS One. 2021 Sep 23;16(9):e0250395. doi: 10.1371/journal.pone.0250395 (PMC8460017; doi:10.1371/journal.pone.0250395)
Supplement: S1 Table — Effect size (Est.) and 95% confidence interval (95% CI) estimates between comparison samples are shown. P-values (p) presented describe the likelihood of statistical difference with values < 0.05 presented in bold. (DOCX) [file pone.0250395.s002.docx]

**Supplemental Table 1.** Model estimates testing for differences in phloem and bark lignin concentrations (g/mg FW) among sample sites of *P. flexilis,* *P. longaeva*, and *P. balfouriana* (see Table 1, Fig. 1). Effect size (Est.) and 95% confidence interval (95% CI) estimates between comparison samples are shown. P-values (p) presented describe the likelihood of statistical difference with values < 0.05 presented in bold.

|  | Phloem | | Bark | |
| --- | --- | --- | --- | --- |
| Within species  Population comparisons | Est. (95% CI) | p | Est. (95% CI) | p |
| *P. flexilis* |  |  |  |  |
| Ruby vs White | 0.57 (-0.57, 1.71) | 0.542 | 15.2 (1.09, 29.2) | **0.030** |
| Snake vs White | 0.36 (-0.78, 1.51) | 0.825 | 8.29 (-5.77, 22.4) | 0.394 |
| Sierra vs White | -0.06 (-1.15, 1.04) | 0.999 | 10.3 (-3.19, 23.8) | 0.185 |
| Snake vs Ruby | -0.20 (-0.20, -1.14) | 0.933 | -6.86 (-18.3, 4.62) | 0.383 |
| Sierra vs Ruby | -0.62 (-1.50, 0.25) | 0.238 | -4.84 (-15.6, 5.96) | 0.623 |
| Sierra vs Snake | -0.42 (-1.30, 0.46) | 0.575 | 2.02 (-8.77, 12.8) | 0.957 |
| *P. longaeva* |  |  |  |  |
| Ruby vs White | 0.35 (-0.05, 0.76) | 0.095 | 9.99 (-2.20, 22.2) | 0.126 |
| Snake vs White | 0.23 (0.17, 0.63) | 0.348 | 2.80 (-9.40, 15.0) | 0.842 |
| Snake vs Ruby | -0.12 (0.52, 0.27) | 0.734 | -7.20 (-19.1, 4.75) | 0.315 |
| *P. balfouriana* |  |  |  |  |
| Sierra vs Klamath | 0.15 (-0.03, 0.34) | 0.101 | -1.81 (-9.43, 5.81) | 0.627 |
